# From complexity to simplicity: a traditional-inspired roasting-sealing process enhances jujube aroma and antioxidant properties

**DOI:** 10.1016/j.fochx.2026.104109

**Published:** 2026-06-15

**Authors:** Shan Tian, Yudie Yao, Changlai Liu, Benliang Deng

**Affiliations:** a Life Science College, Luoyang Normal University, Luoyang 471934, Henan, China; b Bamboo Research Institute, Nanjing Forestry University, Nanjing 210037, Jiangsu, China

**Keywords:** Aroma enhancement, Jujube fruit, Metabolomics, Processing simplification, Traditional food

## Abstract

Smoked jujube (“Wu Zao”), a time-honored Chinese delicacy crafted through generations of drying and smoking, is a laborious process. This study simplified the process into a two-step method: dry-heat roasting (190 °C, 2 min) followed by sealed aging (14 d). Four groups were compared: control (CK), sealed-only (CK-S), roasted-only (CK-R) and combined treatment (JX). Compared with others, JX showed superior flavor attributes, with higher antioxidant compound accumulation (e.g., phenolics: 13.2 vs 9.2 mg/g DW). Strong Maillard reaction was evident from 5-hydroxymethylfurfural (∼17-fold increase). Metabolomics identified 854 differential metabolites, including upregulated flavor-active compounds (e.g., 6-gingerol) and antioxidants (e.g., ferulic acid). Compared with traditional Wu Zao, JX showed a closely comparable overall sensory profile, but with stronger aroma and higher antioxidant capacity, despite lower sweetness and softness. This work demonstrates that roasting-sealed aging synergy induces chemical transformation beyond preservation, offering a practical strategy for nutritious, flavor-rich fruit products.

## Highlights

• Simplified two-step protocol mimics traditional smoked jujube processing.
• Brief high-temperature roasting triggers intense Maillard reactions, boosting aroma.
• Combined treatment synergistically enhances antioxidant capacity.
• Sealed aging amplifies roasting-generated flavors, yielding flavor-enhanced jujube.

## Graphical abstract

**Figure:**
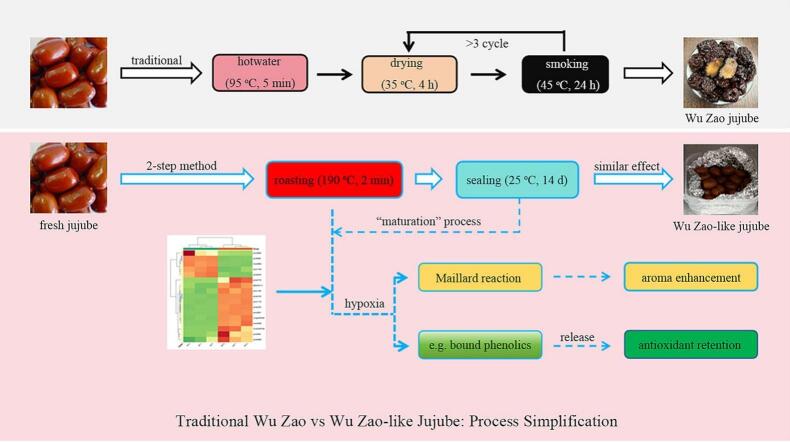

## 1 Introduction

Jujube (*Ziziphus jujuba* Mill.) is a nutritious fruit rich in bioactive compounds (Bai et al., 2016; Cai et al., 2024; Wang et al., 2016), contributing to its high nutritional and medicinal value. However, its high moisture content and active metabolism make it highly perishable at room temperature. This leads to rapid quality deterioration and susceptibility to microbial infections, resulting in a short shelf-life and significant economic loss (Muqaddas et al., 2025). Ancient Chinese people developed methods to preserve jujube fruits by processing them into delicacies like smoked jujube (Liu et al., 2020; Wu et al., 2025), which not only creates unique flavors but also enables long-term storage.

Inspired by the traditional “smoked jujube” (Wu Zao) production—which typically begins with a brief blanching in 90–100 °C hot water for approximately 3–5 min, followed by repeated cycles of drying (30–35 °C for 2–4 h), low-temperature smoking (30–45 °C with wood fire for 12–24 h), re-drying, and a second smoking stage (35–50 °C for 8–16 h), with this drying-smoking cycle repeated 3–5 times—this traditional practice ultimately yields a uniquely flavored delicacy with dark black appearance, rich smoky aroma, soft glutinous texture, and high sugar content (Anonymous., 1974). We hypothesized that the repeated drying and smoking cycles in traditional Wu Zao processing promote Maillard reactions, which are primarily responsible for generating the characteristic smoky aroma (Starowicz & Zieliński, 2019; Van Boekel, 2006). Based on this hypothesis, we proposed that a simplified treatment—brief high-temperature dry-heat roasting (to simulate the drying/smoking) combined with sealed storage—might achieve similar aroma enhancement while dramatically reducing processing complexity (Agila & Barringer, 2012).

To address the practical limitations of traditional Wu Zao processing, which is labor-intensive and time-consuming (Wu et al., 2025), we simplified the process into a two-step protocol: (1) brief high-temperature dry-heat roasting to achieve initial drying, sterilization, and a foundation for potential aroma formation; and (2) sealed storage for anaerobic aging to investigate whether this combined treatment can enhance aroma like traditional Wu Zao. This simplified approach reduces repeated high-temperature/long-duration treatments that can cause significant degradation of bioactive compounds, potentially retaining higher nutritional value and offering a more controllable, industrially viable strategy. Dry-heat treatment can reduce moisture, inhibit microbial infection, and induce Maillard reactions (Agila & Barringer, 2012). Sealed storage creates a modified atmosphere (MAP) environment that suppresses respiration and microbial growth (Soltani et al., 2015; Wang et al., 2025; Zhang et al., 2015).

While the individual effects of heat treatment and MAP on postharvest preservation have been studied (Morgado et al., 2016; Rodov et al., 2000), significant research gaps remain. The application of brief, high-temperature dry-heat roasting as a standalone pretreatment for fresh fruit, particularly in synergy with sealed storage at ambient temperature, is largely unexplored. Furthermore, the potential of this specific combination (dry-heat + sealed storage) to actively drive desirable biochemical transformations—akin to traditional aging processes—toward enhanced flavor and nutritional quality remains unknown.

Therefore, the present study aims to verify the aforementioned hypothesis by combining dry-heat roasting with subsequent sealed storage. We employed an integrated approach combining physiological and biochemical assays with widely targeted metabolomics to evaluate this combined treatment through comprehensive comparisons among all treatment groups. Metabolomics serves to illuminate the metabolic basis underlying the quality of the final product relative to the untreated control, providing a scientific basis for modernizing traditional fruit processing into a controllable, value-adding strategy.

## 2 Materials and methods

### 2.1 Materials

#### 2.1.1 Plant material

Fresh jujube fruits (*Ziziphus jujuba* Mill. cv. ‘Jinsixiaozao’) at commercial maturity were purchased from a local supermarket. Approximately 5 kg of fruits were selected based on uniformity in size, color, and absence of physical damage or disease. The selected fruits were first surface-sterilized by immersion in a 1% (*v*/v) sodium hypochlorite solution for 5 min, followed by rinsing with distilled water and air-drying at room temperature.

#### 2.1.2 Chemicals and reagents

The reagents used for physiological, biochemical, and metabolomic analyses were of analytical or HPLC grade. Key reagents included: methanol (HPLC grade, for metabolite extraction), acetonitrile and formic acid (LC-MS grade, for mobile phase), 2-chloro-*L*-phenylalanine (internal standard for metabolomics, purity 98%), Folin-Ciocalteu reagent, sodium carbonate (Na₂CO₃), aluminum chloride (AlCl₃), sodium nitrite (NaNO₂), and sodium hydroxide (NaOH) for phenolic and flavonoid assays. 2,4,6-Tris(2-pyridyl)-*s*-triazine (TPTZ) and Iron(III) chloride (FeCl₃) were used for FRAP assay. 3,5-Dinitrosalicylic acid (DNS) reagent was used for reducing sugar determination. Ninhydrin reagent was used for free amino acid quantification. 2,6-Dichlorophenolindophenol (DCPIP) was used for Vitamin C determination. Standard salt solutions were used for water activity meter calibration.

### 2.2 Experimental design

The fresh fruits were randomly divided into four treatment groups to investigate the individual and combined effects of dry-heat roasting and sealed storage:

(1) Control Group (CK): Fruits placed in ventilated plastic boxes and stored at room temperature (25 ± 2 °C) in the dark for 14 days.
(2) Sealed Control Group (CK-S): Fruits sealed in airtight plastic boxes (with silicone sealing gaskets) and stored at room temperature (25 ± 2 °C) in the dark for 14 days.
(3) Roasted Group (CK-R): Fruits were evenly spread on a baking tray and roasted in a preheated household oven at 190 ± 5 °C for 2 min. After cooling, they were placed in ventilated plastic boxes and stored under the same conditions as the CK group for 14 days.
(4) Combined Treatment Group (JX): Fruits underwent the same roasting process as the CK-R group. After cooling, they were sealed in airtight plastic boxes and stored at room temperature (25 ± 2 °C) in the dark for 14 days (Table S1).

The processing parameters (190 °C for 2 min) were determined based on preliminary experiments balancing processing efficiency and controllability. Samples were collected at day 0 (immediately after treatment), day 1, and day 14. For widely targeted metabolomics, only day 14 samples from CK and JX groups were selected. Each group at each time point consisted of three independent biological replicates (approximately 15 fruits per replicate).

### 2.3 Physicochemical and bioactive compound analysis

#### 2.3.1 Microscopy and physicochemical analysis

Microscopic analysis was conducted on jujube tissue sections using a Nikon Eclipse E200 optical microscope. Thin sections (≈50 μm) were manually prepared from the mesocarp (flesh) tissue with a surgical blade, placed on slides, and observed unstained under a 10× objective to evaluate cellular integrity.

Key physicochemical properties were characterized. Surface color was measured with a chroma meter in CIELAB coordinates (L*, a*, b*) (Kaur et al., 2022). Texture Profile Analysis determined hardness (peak force in N) (Kaur et al., 2022; Rivero et al., 2021). Water activity (a_w) was measured with a calibrated water activity meter (AW-1, Rotronic, Switzerland) using standard salt solutions for calibration prior to measurement. Soluble solids content (°Brix) was determined using a digital refractometer (Kaur et al., 2022). Acidity was assessed via pH measurement and titratable acidity (TA) expressed as percentage of malic acid equivalent (Rivero et al., 2021).

#### 2.3.2 Bioactive compound analysis

Bioactive compounds were quantified. Total antioxidant capacity was determined by FRAP assay, which measures the reduction of Fe^3+^-TPTZ complex to Fe^2+^-TPTZ at low pH, with absorbance measured at 593 nm (Benzie & Strain, 1996). Total phenolic content was measured using the Folin-Ciocalteu method, based on the reduction of phosphomolybdic/phosphotungstic acid complexes, with absorbance measured at 765 nm (Singleton & Rossi, 1965). Total flavonoids were analyzed via AlCl₃ colorimetry, where flavonoids form complexes with AlCl₃, measured at 510 nm (Matić et al., 2017). Vitamin C was determined by DCPIP titration, based on the reduction of the blue dye 2,6-dichlorophenolindophenol by ascorbic acid. 5-Hydroxymethylfurfural (5-HMF) content was measured spectrophotometrically at 284 nm (Adu et al., 2019). Free amino acids were quantified using the ninhydrin assay, where amino acids react with ninhydrin to form a purple-colored product, measured at 570 nm (Rosen, 1957). Reducing sugars were analyzed via the DNS method, where reducing sugars reduce 3,5-dinitrosalicylic acid to orange-colored products, measured at 540 nm (Blakeney & Mutton, 1980).

#### 2.3.3 Statistical analysis

Data were analyzed using SPSS software (Version 21.0, IBM Corp., USA). Differences between treatment groups across different storage time points were evaluated using two-way ANOVA, with treatment and storage time as the two main factors, followed by Duncan's multiple range test at a significance level of *p* < 0.05. Significant differences among treatment groups at each time point are denoted by different lowercase letters in the figures. Each treatment and sampling time point consisted of three independent biological replicates.

### 2.4 Sensory evaluation

Sensory evaluation was performed by a trained panel (*n* = 10, comprising graduate students and faculty members from the Department of Food Science, ages 22–45 years). Prior to formal evaluation, the panel participated in three training sessions (1 h each) over one week to familiarize themselves with the sensory attributes and rating scales. During formal evaluation, samples were presented in randomized order in white plastic trays labeled with random three-digit codes. Quantitative descriptive analysis was used, and key attributes (taste, texture, flavor, aroma) were rated on intensity scales (0–9), culminating in an overall acceptability score on a 9-point hedonic scale (1 = extremely dislike, 9 = extremely like). Evaluations were conducted in a sensory laboratory with individual booths under white fluorescent lighting. Sensory data were analyzed using ANOVA followed by Duncan's multiple range test (*p* < 0.05) to determine significant differences among treatment groups (Kaur et al., 2022; Rivero et al., 2021).

### 2.5 *Metabolomics analysis*

#### 2.5.1 Sample preparation and metabolite extraction

For widely targeted metabolomics analysis, the processed jujube fruit tissue samples were initially freeze-dried using a vacuum freeze-dryer (Scientz-100F, China) and then ground into a homogeneous fine powder with a grinder (MM 400, Retsch) at 30 Hz for 1.5 min, and passed through a 0.5 mm sieve to ensure uniform particle size. Following the established and optimized protocol described by Zou et al. (2020), precisely 30 mg of the powder was weighed and extracted with 1500 μL of a pre-cooled (−20 °C) 70% methanol aqueous solution containing the internal standard 2-Chloro-*L*-phenylalanine (1 ppm, purity 98%, J&K Scientific, lot #106151, CAS: 103616-89-3) for process monitoring. The internal standard was used solely for quality control to monitor instrument stability and was not used for data normalization. After thorough extraction via intermittent vortex mixing (30 s every 30 min, repeated six times), the mixture was centrifuged at 12,000 rpm for 3 min. The supernatant was carefully collected, passed through a 0.22 μm microporous membrane filter, and aliquoted into injection vials for subsequent ultra-performance liquid chromatography-tandem mass spectrometry analysis.

#### 2.5.2 UPLC-ESI-MS/MS analysis

Widely targeted metabolomic profiling was performed by Wuhan Metware Biotechnology Co., Ltd. (http://www.metware.cn) on an ultra-performance liquid chromatography-tandem mass spectrometry system (UPLC, ExionLC™ AD; MS, QTRAP® 6500+, Sciex). Chromatographic separation was performed on an Agilent SB-C18 column (1.8 μm, 2.1 mm × 100 mm) maintained at 40 °C. The mobile phase consisted of solvent A (water with 0.1% formic acid) and solvent B (acetonitrile with 0.1% formic acid), eluted under the following gradient program: starting at 95% A, linearly decreasing to 5% A over 9.0 min, holding for 1.0 min, and then re-equilibrating to initial conditions over 3.0 min, with a constant flow rate of 0.35 mL/min. The mass spectrometer was operated with the electrospray ionization source temperature set to 500 °C and ion spray voltages of 5500 V (positive) and − 4500 V (negative). Gas parameters were configured as follows: nebulizer gas (GSI) 50 psi, auxiliary gas (GSII) 60 psi, curtain gas (CUR) 25 psi, with collision gas set to medium. Data acquisition was performed in multiple reaction monitoring mode, with declustering potential and collision energy individually optimized for each metabolite transition (Zou et al., 2020). To monitor instrument stability and data quality, quality control (QC) samples were prepared by pooling equal volumes of extracts from all samples. A QC sample was injected at the beginning of the run for column equilibration and then every 6 sample injections throughout the analytical sequence. The stability of the analytical platform was evaluated by examining the total ion current (TIC) chromatograms, retention time reproducibility, and coefficient of variation (CV) of detected peaks in QC samples.

#### 2.5.3 Metabolomic data analysis

Raw data from UPLC-MS/MS were processed to construct a data matrix of metabolite peak areas. Unsupervised principal component analysis was performed on unit variance (UV) scaled data using the prcomp function in R. For data normalization, UV scaling (also known as *Z*-score standardization) was applied, where each variable was centered by subtracting its mean and scaled by dividing by its standard deviation, resulting in a mean of 0 and standard deviation of 1. To enhance group discrimination and identify key differential metabolites, supervised orthogonal projections to latent structures-discriminant analysis was further applied using the R package MetaboAnalystR, with data log2-transformed and mean-centered (zero-centered) prior to analysis; model validity was confirmed through a permutation test (*n* = 200). Differential metabolites between groups were identified by applying a combined threshold of variable importance in projection score > 1 from the OPLS-DA model, absolute log2(fold change) ≥ 1.0, and statistical significance (*P*-value <0.05 or FDR < 0.05). Hierarchical cluster analysis and Pearson correlation coefficient calculations were conducted and visualized as heatmaps using the R package ComplexHeatmap. Metabolite annotation was performed based on the KEGG Compound database, and it should be noted that all metabolites reported are tentatively annotated based on MS/MS spectral matching unless explicitly confirmed with authentic standards, followed by KEGG pathway enrichment analysis on the differential metabolites to elucidate significantly altered biological pathways (Zou et al., 2020).

## 3 **Results**

### 3.1 Phenotypic and cellular structural changes in processed jujube fruits

**Fig. 1:**
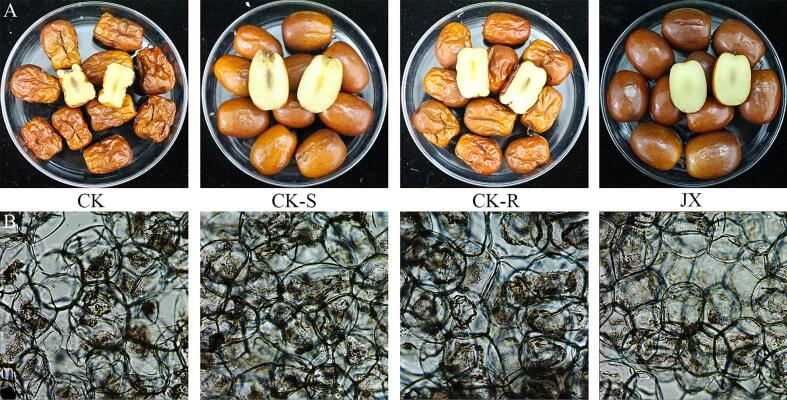
Macroscopic phenotype (A) and cellular integrity (B, 10×) after 14 days. Scale bar = 100 μm. Abbreviations: CK, control (ventilated at 25 ± 2 °C); CK-S, sealed; CK-R, roasted (190 °C, 2 min, then ventilated); JX, roasted then sealed.

Based on the phenotypic observations and microstructural analysis presented in Fig. 1a, distinct visual and cellular changes were evident among the four jujube samples after 14 days of storage. Overall, phenotypically, all groups retained the characteristic red color of jinsixiaozao, but with noticeable variations in hue and intensity. The low-moisture nature of the ‘Jinsixiaozao’ cultivar (≈15–20% fresh weight) inherently resisted microbial growth; no visible mold contamination was observed in any group during the 14-day storage, although the unsealed control (CK) and sealed-only group (CK-S) showed evident senescence, while the roasted groups (CK-R and JX) maintained better structural integrity. Distinct visual differences were observed among the treatments. The control (CK) group showed severe surface wrinkling, a shrunken morphology, and the darkest brown color, with dark, compact flesh. Sealing alone (CK-S) moderately improved appearance, with reduced wrinkling and a slightly lighter color; its flesh was more uniform. Dry-heat roasting (CK-R) yielded fuller fruits with minimal wrinkling, a uniform medium-brown peel, and lighter, looser internal tissue. The combined treatment (JX) produced the optimal appearance: plump, almost smooth fruits with a bright, uniform color. Their cross-section revealed the lightest (pale yellow), porous, and well-structured flesh. Overall, a clear gradient from CK to JX was evident: progressive reduction in shrinkage and wrinkling, gradual lightening of peel and flesh color, and development of a looser, more porous texture. The JX protocol most effectively preserved fresh-like turgor and desirable visual quality.

The optical microscopy analysis revealed a clear hierarchy in cellular integrity among the four groups after 14 days of storage (Fig. 1b). The JX group exhibited the clearest cell boundaries, intact structure without significant damage, and regular morphology. The CK-S and CK-R groups maintained relatively complete cellular structures with clear boundaries, though slight deformation was observed in some cells. In contrast, the most severe disruption occurred in the CK group, where cellular boundaries were nearly absent, content loss was extensive, and cells appeared disintegrated.

Based on the comprehensive evaluation of physicochemical and sensory attributes after 14 days of storage (Table 1), the four postharvest treatments resulted in jujube products with distinct quality profiles. Notably, while the roasting process effectively reduces moisture initially, no significant difference in water activity (aw) was observed between the roasted-only group (CK-R, 0.68) and the control (CK, 0.72) after 14 days of storage (Table 1). The combined treatment group (JX), designed to produce the target aroma-enhanced Jujube, demonstrated the most favorable overall characteristics, particularly excelling in color and consumer acceptance.

**Table 1:** Attributes of jujube products after 14 days of storage. Data are presented as mean ± SD (n = 3). Different letters indicate significant differences (p < 0.05, Duncan's test). Abbreviations: CK, control (ventilated storage at 25 ± 2 °C); Sealing (sealed in airtight boxes); Roasting (190 °C, 2 min, then ventilated); JX (190 °C, 2 min, then sealed); L*, a*, b*, CIELAB color coordinates; a_w, water activity; °Brix, soluble solids; TA, titratable acidity (%); N, hardness.

Parameter	CK group	Sealing group	Roasting group	JX group
Surface Color (lightness, L*)	52.3 ± 1.2a	48.7 ± 1.5b	45.2 ± 1.1c	43.8 ± 0.9c
Surface Color (redness, a*)	8.2 ± 0.5d	12.5 ± 0.8c	14.8 ± 0.6b	16.3 ± 0.4a
Surface Color (yellowness, b*)	28.6 ± 1.3a	25.4 ± 1.1b	22.1 ± 0.8c	20.5 ± 0.7c
Texture Hardness (N)	25.6 ± 1.1b	18.3 ± 0.9d	32.7 ± 1.7a	20.9 ± 1.3c
Water Activity (a_w)	0.72 ± 0.02c	0.85 ± 0.04a	0.68 ± 0.03c	0.78 ± 0.03b
Soluble Solids Content (^o^Brix)	24.5 ± 1.1b	22.8 ± 0.5c	28.3 ± 1.2a	26.1 ± 1.4ab
Titratable Acidity (%)	0.38 ± 0.03b	0.52 ± 0.04a	0.31 ± 0.02c	0.48 ± 0.02a
Overall Acceptability Score	3.5 ± 0.3c	5.8 ± 0.4b	6.2 ± 0.3b	7.6 ± 0.2a

The JX group exhibited a distinct surface color, with a redness (a*) value of 16.3, which was significantly higher than that of the control (CK, 8.2) and the sealing-only group (CK-S, 12.5) (*p* < 0.05), representing an increase of approximately 1.99-fold and 1.30-fold, respectively. Concomitantly, the L* value (lightness) decreased from 52.3 in CK to 43.8 in JX, and b* (yellowness) decreased from 28.6 to 20.5, indicating a shift toward a deeper, more reddish-brown hue that visually resembles the appearance of traditional Wu Zao (black smoked jujube), albeit less intense. This distinct color, likely enhanced by both Maillard reaction during roasting and preservation under sealed conditions, contributed significantly to its visual appeal. In terms of texture, the JX group showed a balanced hardness (20.9 N), being 18% softer than the control (25.6 N) and 36% softer than the excessively hard roasted-only group (32.7 N), suggesting a more palatable mouthfeel. Notably, when compared with traditional Wu Zao products from three representative producing regions (Yanan, Dingxi, and Zhongwei), the JX product exhibited significantly higher hardness (20.9 N) than all three Wu Zao samples (range: 14.3–16.1 N), indicating that traditional Wu Zao possesses a softer, more glutinous texture, while JX retains a firmer bite (Table S2). Its water activity (a_w = 0.78) was maintained at a favorable level, being 8% higher than CK (0.72) and 15% higher than the roasted-only group (0.68), indicating better moisture retention which prevented excessive hardening. The soluble solids content in JX (26.1°Brix) was 7% higher than CK (24.5°Brix), contributing to sweetness, while its titratable acidity (0.48%) was 26% higher than CK (0.38%), providing a pleasant sweet-tart balance. Most conclusively, the JX group received the highest overall acceptability score of 7.6, which was 117% higher than the control (3.5) and 23% higher than the roasted-only group (6.2). This superior score underscores that the synergistic effect of roasting followed by sealed storage (JX) optimally enhanced key sensory attributes—color, texture, and flavor balance—resulting in a product that was significantly more preferred by consumers compared to all other treatments.

A preliminary comparison between the JX product and commercially available traditional Wu Zao from three representative producing regions (Yanan, Dingxi, and Zhongwei) was conducted to contextualize the product attributes (Table S2). Notably, the JX product exhibited significantly higher antioxidant capacity (FRAP value: 8.7 vs. 2.4–2.8 mmol Fe^2+^/g DW) compared to all traditional Wu Zao samples, indicating superior nutritional value. In terms of texture, the JX product showed significantly higher hardness (20.9 N) than all three Wu Zao samples (range: 14.3–16.1 N), indicating that traditional Wu Zao possesses a softer, more glutinous texture, while JX retains a firmer bite. Regarding overall acceptability, the JX product scored 7.6, which was slightly lower than traditional Wu Zao samples (8.2–8.4), suggesting that the traditional product remains marginally preferred by consumers, possibly due to its softer texture and characteristic black color. However, the JX process follows a simple two-step protocol (roasting + sealing), whereas the traditional Wu Zao method is highly complex and labor-intensive.

### 3.2 Effects of processing and storage on antioxidant accumulation in jujube products

**Fig. 2:**
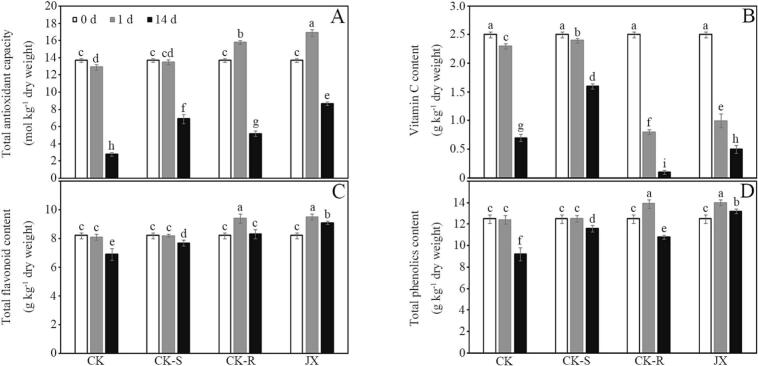
Antioxidant capacity and bioactive compounds. Changes in antioxidant capacity and bioactive compounds in jujube fruits during 14 days of storage. (A) Total antioxidant capacity. (B) Vitamin C content. (C) Total flavonoid content. (D) Total phenolic content. Data are presented as mean ± SD (*n* = 3). Different letters above the bars indicate significant differences among treatment groups at each specific time point according to Duncan's multiple range test (*p* < 0.05). The x-axis shows the four treatment groups: CK (control), CK-S (sealed), CK-R (roasted), and JX (roasting + sealed).

The FRAP assay, reflecting total antioxidant capacity, showed distinct changes across treatments and storage time (Fig. 2a). On day 0, all groups shared a baseline value of 13.7 mmol Fe^2+^/g DW. After one day, the control (CK) decreased slightly to 12.9 mmol Fe^2+^/g DW (−5.8%), while the sealed group (CK-S) remained relatively stable at 13.5 mmol Fe^2+^/g DW (−1.5%). In contrast, both heat-treated groups exhibited a marked increase: the roasted-only group (CK-R) rose to 15.8 mmol Fe^2+^/g DW (+15.3%), and the combined roasted-and-sealed group (JX) reached 16.9 mmol Fe^2+^/g DW (+23.4%). By day 14, all groups showed a decline from day 0, but the extent varied greatly. CK dropped sharply to 2.8 mmol Fe^2+^/g DW (−79.6%), CK-S declined to 6.9 mmol Fe^2+^/g DW (−49.6%), and CK-R fell to 5.2 mmol Fe^2+^/g DW (−62.0%). Notably, the JX group retained the highest FRAP value at 8.7 mmol Fe^2+^/g DW, representing a − 36.5% change from day 0 and a value substantially higher than CK, CK-S, and CK-R at this time point. This indicates that the combined roasting and sealing treatment (JX) was most effective in mitigating the long-term loss of antioxidant capacity, likely due to the initial heat-induced formation of antioxidants and the subsequent sealed storage slowing their degradation.

Vitamin C content was highly sensitive to both heat treatment and storage conditions (Fig. 2b). The initial content was 2.5 mg/g DW for all groups. After one day, CK and CK-S showed minor reductions to 2.3 mg/g DW (−8.0%) and 2.4 mg/g DW (−4.0%), respectively. However, the heat-treated groups suffered severe losses: CK-R decreased to 0.8 mg/g DW (−68.0%) and JX to 1.0 mg/g DW (−60.0%). This demonstrates the acute destructive effect of the 190 °C roasting on this heat-labile compound. After 14 days of storage, VC content further degraded in all groups. CK dropped drastically to 0.7 mg/g DW (−72.0% from day 0), while CK-S retained a higher level at 1.6 mg/g DW (−36.0%). The roasted groups had minimal residual VC: CK-R at 0.1 mg/g DW (−96.0%) and JX at 0.5 mg/g DW (−80.0%). Although sealing alone (CK-S) helped preserve VC compared to the open-air control, the initial thermal degradation in JX was so profound that even subsequent sealed storage could not compensate, resulting in the lowest final VC content among non-roasted groups. The superior sensory quality of JX group is thus not attributable to VC retention.

Total flavonoid content exhibited a more stable or even increasing trend, particularly in response to heat (Fig. 2c). The baseline TFC was 8.2 mg CE/g DW. On day 1, CK and CK-S remained nearly unchanged at 8.1 mg CE/g DW (−1.2%) and 8.2 mg CE/g DW (0.0%), respectively. In contrast, roasting induced an immediate increase: CK-R rose to 9.4 mg CE/g DW (+14.6%) and JX to 9.5 mg CE/g DW (+15.9%). This suggests that short-term high-temperature treatment may facilitate the release or conversion of bound flavonoids. After 14 days, a gradual decline was observed in the non-roasted or roasted-only groups: CK decreased to 6.9 mg CE/g DW (−15.9%), CK-S to 7.7 mg CE/g DW (−6.1%), and CK-R to 8.3 mg CE/g DW (+1.2% from day 0). Remarkably, the JX group not only maintained but slightly increased its day-1 gain, reaching 9.1 mg CE/g DW by day 14, which is +11.0% higher than the initial level. This final TFC in JX was significantly higher than in CK (9.1 vs. 6.9 mg CE/g DW), CK-S (9.1 vs. 7.7 mg CE/g DW), and CK-R (9.1 vs. 8.3 mg CE/g DW). The combined roasting and sealing protocol effectively enhanced and preserved flavonoid content, which may contribute to the antioxidant activity and distinctive flavor profile of the final aroma-enhanced Jujube.

Similar to TFC, total phenolic content responded positively to heat treatment (Fig. 2d). The starting value was 12.5 mg GAE/g DW for all samples. After one day, CK and CK-S showed negligible change (12.4 mg GAE/g DW, −0.8% and 12.5 mg GAE/g DW, 0.0%). The roasted groups, however, showed a clear increase: CK-R increased to 13.9 mg GAE/g DW (+11.2%) and JX to 14.0 mg GAE/g DW (+12.0%). This indicates that thermal processing promoted the extraction or formation of phenolic compounds. During the 14-day storage, phenolic content declined across all groups but to varying degrees. CK experienced the largest drop to 9.2 mg GAE/g DW (−26.4%). Sealing alone (CK-S) offered some protection, resulting in a final content of 11.6 mg GAE/g DW (−7.2%). The roasted-only group (CK-R) decreased to 10.8 mg GAE/g DW (−13.6%). Importantly, the JX group retained the highest phenolic content at 13.2 mg GAE/g DW, which is only −5.6% lower than its day-1 value and + 5.6% higher than the original day-0 level. Compared to day 14 controls, JX's TPC was 43.5% higher than CK, 13.8% higher than CK-S, and 22.2% higher than CK-R. This demonstrates that the synergistic effect of initial roasting and subsequent sealed storage was highly effective in preserving phenolic compounds, which likely plays a crucial role in the enhanced antioxidant capacity (FRAP) and the development of the desirable sensory attributes in aroma-enhanced Jujube.

### 3.3 Roasting and sealing effects on flavor chemistry in jujube fruits

**Fig. 3:**
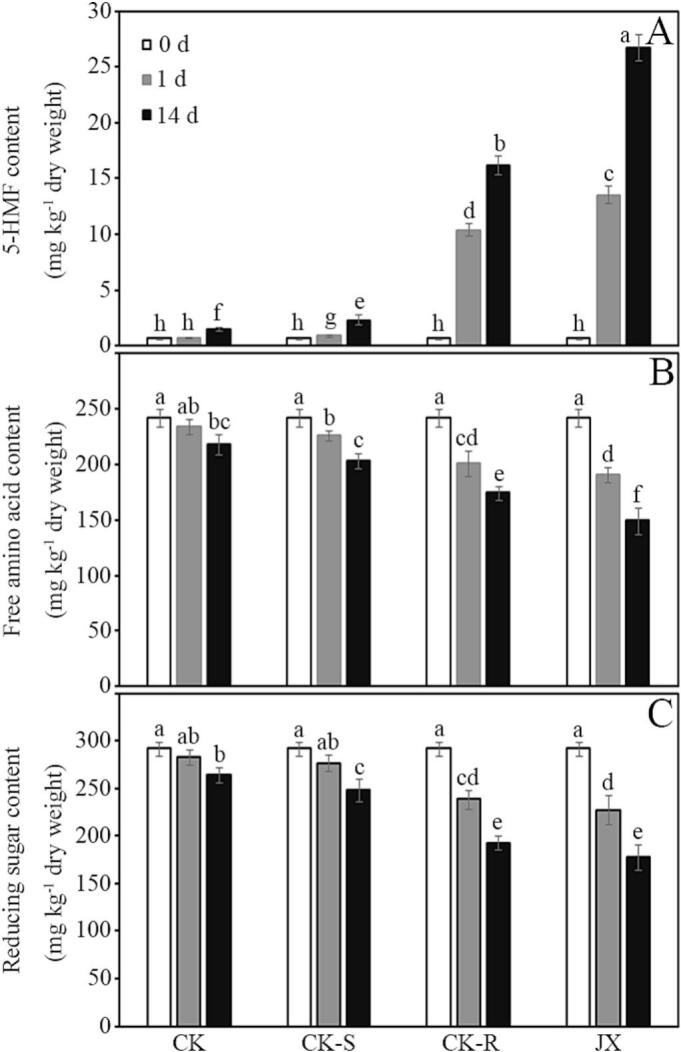
(A) 5-HMF content. (B) Free amino-acid content. (C) Reducing-sugar content. Data are presented as mean ± SD (n = 3). Different letters above the bars indicate significant differences among treatment groups at each specific time point according to Duncan's multiple range test (p < 0.05). The x-axis shows the four treatment groups: CK (control), CK-S (sealed), CK-R (roasted), and JX (roasting + sealed). Abbreviations: 5-HMF, 5-hydroxymethylfurfural.

Sensory evaluation was performed to complement the biochemical and metabolomic analyses. The 5-HMF content, a key indicator of Maillard reaction and thermal processing, showed dramatic increases in response to roasting (Fig. 3a). All groups started at an identical baseline of 0.67 mg/kg DW. After one day, the control (CK) and sealed-only group (CK-S) showed only minor increases to 0.81 mg/kg DW (+20.9%) and 0.93 mg/kg DW (+38.8%), respectively. In stark contrast, roasting triggered a substantial surge: the roasted-only group (CK-R) increased to 10.45 mg/kg DW (+1460%), and the combined roasted-and-sealed group (JX) reached an even higher 13.56 mg/kg DW (+1924%). This confirms that the 190 °C heat treatment was the primary driver for 5-HMF formation. After 14 days of storage, levels continued to rise in all groups. CK and CK-S reached 1.58 mg/kg DW (+136%) and 2.37 mg/kg DW (+254%), respectively. The roasted groups saw further significant accumulation: CK-R increased to 16.23 mg/kg DW (+2322% from day 0), while the JX group exhibited the highest final concentration at 26.73 mg/kg DW (+3890%). The final 5-HMF content in JX was approximately 17-fold higher than in CK. This pronounced accumulation in JX, resulting from the combination of initial heat-induced generation and subsequent promotion under sealed, anaerobic storage conditions, is strongly correlated with the development of the characteristic caramel-like and roasted aroma in the aroma-enhanced Jujube.

The total free amino acid content, serving as crucial precursors for the Maillard reaction, exhibited a consistent declining trend throughout storage, accelerated by heat treatment (Fig. 3b). The initial content was 242 mg/kg DW for all samples. After one day, a slight decrease was observed in CK (234 mg/kg DW, −3.3%) and CK-S (226 mg/kg DW, −6.6%). The decrease was more pronounced in the roasted groups: CK-R fell to 201 mg/kg DW (−16.9%) and JX to 191 mg/kg DW (−21.1%), indicating significant consumption of amino acids during the thermal reaction. This trend continued over the 14-day storage period. CK declined to 218 mg/kg DW (−9.9% from day 0), and CK-S to 203 mg/kg DW (−16.1%). The roasted groups showed the greatest depletion: CK-R decreased to 174 mg/kg DW (−28.1%) and, most notably, the JX group reached the lowest final level of 149 mg/kg DW (−38.4%). The final FAA content in JX was 31.7% lower than in CK. This substantial reduction in the JX group highlights the intensive utilization of free amino acids, which, in conjunction with reducing sugars, participated in advanced Maillard reaction pathways during the extended sealed storage period following roasting. This consumption is a key biochemical process underpinning the generation of the complex, savory, and roasted flavor notes characteristic of the aroma-enhanced Jujube.

The reducing sugar content, another essential reactant in the Maillard reaction, demonstrated a pattern of consumption similar to that of free amino acids (Fig. 3c). The starting level was 292 mg/kg DW. After one day, non-roasted groups showed minor reductions: CK decreased to 283 mg/kg DW (−3.1%) and CK-S to 277 mg/kg DW (−5.1%). The roasted groups, however, experienced a marked drop: CK-R fell to 239 mg/kg DW (−18.2%) and JX to 228 mg/kg DW (−21.9%), directly linking significant sugar consumption to the heat treatment. During the subsequent 14-day storage, the decline continued. CK and CK-S reached 265 mg/kg DW (−9.2%) and 249 mg/kg DW (−14.7%), respectively. The reduction was most severe in the heat-treated groups: CK-R declined to 193 mg/kg DW (−33.9%), and the JX group exhibited the lowest final content at 178 mg/kg DW (−39.0% from day 0). The final RS content in JX was 32.8% lower than in CK. This pronounced and progressive decrease in reducing sugars within the JX group, especially during the sealed aging phase, indicates their active and sustained participation in Maillard reaction and possibly caramelization processes. The co-depletion of both reducing sugars and free amino acids (FAA) in the JX treatment provides direct chemical evidence for the enhanced flavor development, confirming that the combined roasting and sealed aging protocol optimally drives the reactions responsible for generating the desired rich, caramelized aroma and taste profile in JX group.

### 3.4 Comprehensive Metabolomic profiling of processed jujube products

#### 3.4.1 Metabolic characterization of processed jujube fruit tissue

**Fig. 4:**
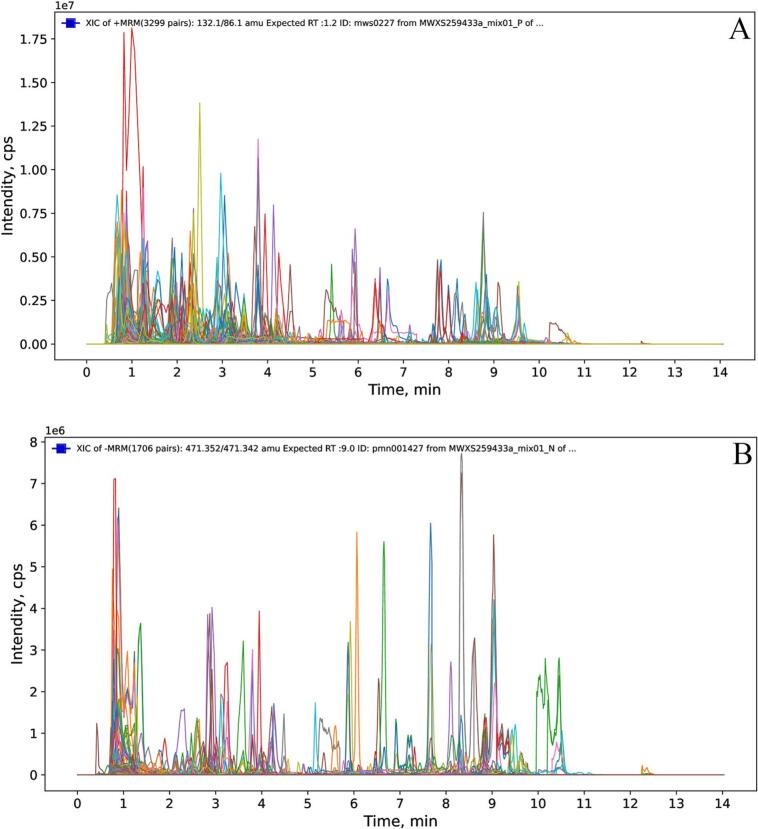
**Untargeted metabolomics analysis of jujube candy.** Total ion current (TIC) chromatograms in (A) positive ion mode and (B) negative ion mode.

The reliability of the metabolomic analysis was confirmed by quality control (QC) samples. The total ion current (TIC) chromatograms showed consistent peak intensities and retention times across all runs (Fig. 4a,b). The coefficient of variation (CV) of peak areas in QC samples was <15% for the majority of detected metabolites, indicating good instrument stability and high data quality.

**Fig. 5:**
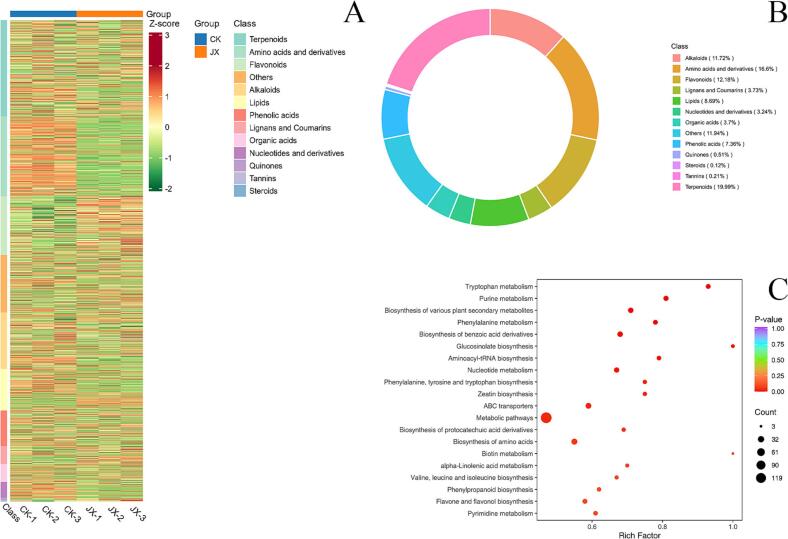
**Metabolite profiling and pathway enrichment of jujube candy versus control.** (A) Heatmap of differentially accumulated metabolites between CK and JX groups. (B) Classification and proportion of identified metabolites in JX. (C) KEGG pathway enrichment analysis of differentially abundant metabolites (dot size = number of metabolites; dot color = *p*-value; redder = more significant). Abbreviations: CK, control group; JX, combined roasting and sealed storage group; KEGG, Kyoto Encyclopedia of Genes and Genomes; DEM, differentially abundant metabolite.

Analysis of the UPLC-ESI-MS/MS data annotated a total of 3301 metabolites, which were categorized into 13 major biochemical classes (Fig. 5a,b). The composition was as follows: 660 terpenoids, 402 flavonoids, 387 alkaloids, 287 lipids, 548 amino acids and derivatives, 243 phenolic acids, 123 lignans and coumarins, 122 organic acids, 107 nucleotides and derivatives, 17 quinones, 7 tannins, 4 steroids, and 394 unclassified compounds. Based on relative abundance, terpenoids constituted the largest proportion (19.99%), followed by amino acids and derivatives (16.6%), flavonoids (12.18%), alkaloids (11.72%), lipids (8.69%), and phenolic acids (7.36%).

The confidence levels of metabolite identification were classified into three levels based on the Metabolomics Standards Initiative (MSI): Level 1 (identified by authentic standards), Level 2 (tentatively identified based on MS/MS spectral matching), and Level 3 (characterized based on physicochemical properties or homologous compounds). The number of metabolites at each confidence level is summarized as follows: Level 1: ∼247 metabolites, Level 2: ∼1523 metabolites, and Level 3: ∼1531 metabolites. The detailed confidence level for each metabolite is provided in Supplementary Table S3.

#### 3.4.2 Multivariate statistical analysis of metabolic profiles

To evaluate global metabolic differences between the control (CK) and combined treatment (JX) groups, we performed multivariate statistical analyses. Both hierarchical clustering and principal component analysis (PCA) demonstrated a clear separation between the CK and JX groups (Fig. S1), indicating distinct metabolite profiles resulting from the treatment. In the PCA model with 95% confidence ellipses (Fig. S1b), the first two principal components explained 68.32% and 9.89% of the total variance, respectively. The non-overlapping ellipses confirmed significant metabolic differences between the two groups and demonstrated the high reproducibility of the metabolomic data.

To further identify metabolites responsible for group discrimination, orthogonal partial least squares-discriminant analysis (OPLS-DA) was applied. Unlike the unsupervised PCA, OPLS-DA uses a supervised approach to maximize separation between predefined groups. The OPLS-DA models successfully distinguished the metabolic profiles of CK and JX samples (Fig. S2), providing a robust basis for identifying differentially abundant metabolites. To validate the reliability of the OPLS-DA model, a permutation test (*n* = 200) was performed. The results demonstrated robust model validity with Q^2^ = 0.989, R^2^X = 0.763, and R^2^Y = 1.0, indicating excellent model fitting and predictive ability without overfitting (Fig. S2b).

#### 3.4.3 Annotation and analysis of differential metabolites

Comparative analysis between JX and CK groups revealed extensive metabolic alterations. Fig. S3 illustrates the volcano plot of all 3301 detected metabolites, showing that 495 metabolites were significantly upregulated, 945 metabolites were significantly downregulated, and 1861 metabolites showed no significant difference in JX compared with CK. By applying a primary screening criterion of Variable Importance in Projection (VIP) ≥ 1 and fold change (FC) ≥ 2 or ≤ 0.5 without FDR filtering, a total of 1440 differential metabolites were identified (Supplementary Table S3). Subsequently, using a more stringent criteria of VIP ≥ 1, FC ≥ 2 or ≤ 0.5, and statistical significance (*P*-value <0.05 or FDR < 0.05), 854 metabolites were annotated as significantly altered, comprising 392 upregulated and 462 downregulated compounds (Table S3). It should be noted that the 1440 metabolites listed in Supplementary Table S3 represent the initial screening set that met the VIP and FC criteria, prior to the application of the statistical significance filter. The 30 metabolites with the most significant changes are listed in Table 2.

**Table 2:** The top 30 metabolites after multiple treatments. Thirty metabolites with the most significant changes were selected (confidence Level 1 or 2, FDR < 0.05, VIP > 1, FC > 2 or FC < 1). Ordered by decreasing FC, they include flavonoid glycosides, coumarins, sesquiterpenoids, and hydroxy-fatty acids, with decreased lysophospholipids, reflecting extensive metabolic remodeling. Abbreviations: VIP, Variable Importance in Projection (OPLS-DA); FC, fold change (JX/CK); FDR, false discovery rate.

Metabolite Name	FC	FDR	VIP	Level	Putative biological significance
(1*R*,3*R*,4*R*,6*S*,7*S*,8*R*)-7,11-dimethyl-4-propan-2-yltricyclo[5.4.0.03,8]undec-10-en-6-ol	13.11	0.0003	1.205	1	stress-responsive metabolite
Isoscopoletin	8.29	0.0030	1.205	1	strong antioxidant and antimicrobial activity
(5*E*,7*E*)-3,8,12-trimethyltrideca-5,7,11-triene-3,4-diol	6.47	0.0034	1.202	1	contributes to fruit aroma and defense
6-Amino-1-naphthoic acid	5.65	0.0107	1.202	1	UV-protective secondary metabolite; antioxidant properties
Macrophypene B	5.06	0.0002	1.205	2	antimicrobial and cytotoxic activities
3-(Cyclohexen-1-yl)-2-hydroxy-4a,5-dimethyl-2,3,4,5,6,7,8,8a-octahydronaphthalen-1-one	5.02	8.9 × 10^−5^	1.205	2	related to plant stress response
[6]-Gingerol	4.95	0.0023	1.199	1	anti-inflammatory and antioxidant
6-Methoxykaempferol-3-O-glucoside	4.86	0.0121	1.195	1	potent antioxidant
6-Hydroxyrhamnocitrin-3-O-glucoside	4.77	0.0228	1.171	1	antioxidant
10-hydroxy-4,6,8,10-tetramethyldodec-4-en-3-one	4.75	0.0168	1.200	1	key volatile contributing to flavor
Benzoate	4.71	0.0193	1.200	2	antimicrobial and preservative
Hispanolone	4.68	0.0009	1.191	2	anti-inflammatory activity
Isorhamnetin-3-O-glucoside	4.42	0.0133	1.163	1	antioxidant
Luteolin 3′-methyl-ether 7,4′-diglucoside	4.09	0.0014	1.172	1	antioxidant and UV-protective
3′-methoxyquercetin-3-O-L-rhamnosyl(1 → 2)glucopyranoside	4.08	0.0027	1.192	1	strong antioxidant
Sexangularetin-3-O-glucoside-7-O-rhamnoside	4.08	0.0107	1.176	1	UV-protective pigment
(9*Z*,11*Z*)-13-hydroxyhexadeca-9,11-dienoic acid	4.04	0.0002	1.204	1	lipid signalling and defense
Tamarixetin-3-O-glucoside-7-O-rhamnoside	3.99	0.0019	1.184	1	antioxidant
Isorhamnetin-3-O-rutinoside	3.74	0.0022	1.184	1	antioxidant
Kaempferol-4′-O-glucoside	3.64	0.0042	1.146	1	antioxidant / anti-inflammatory
Isorhamnetin-3-O-neohesperidoside	3.55	0.0013	1.198	1	antioxidant
Vitexin 7-O-glucoside	3.48	0.0061	1.198	1	neuroprotective and antioxidant
Tamarixetin 7-rutinoside	3.36	0.0034	1.166	1	antioxidant
Kaempferol-3-O-rhamnoside-7-O-glucoside	3.32	0.0021	1.181	1	antioxidant
Nitensoside B	3.21	0.0022	1.181	1	antimicrobial potential
Kaempferol-3-O-glucorhamnoside	3.21	0.0042	1.162	1	antioxidant
Luteolin-7-O-neohesperidoside	3.08	0.0041	1.163	1	antioxidant
Estrane-3,17-diol	2.41	0.0043	1.203	2	possible hormonal regulation
4-Guanidinobutanoate	2.25	0.0090	1.203	1	antimicrobial activity
LPC(18:1/0:0)	0.20	0.0074	1.205	1	lipid mediator

The detailed analysis of differential metabolites further elucidated the specific chemical transformations (Table S3). Regarding primary metabolic precursors, a widespread downregulation of free amino acids was observed, including l-glutamine (VIP = 1.206, FC = 0.22), L-leucine (VIP = 1.199, FC = 0.29), and *L*-phenylalanine (VIP = 1.206, FC = 0.35), indicating their potential consumption in Maillard and Strecker reactions. Several small peptides, however, showed increased levels. Lipid metabolism was also significantly affected, with a pronounced decrease in various lysophospholipids such as lysoPE 16:0 (FC = 0.07) and lysoPC 18:2 (FC = 0.04). Most strikingly, the treatment strongly enhanced the accumulation of numerous flavonoid glycosides and phenolic acids, including apigenin-7-O-rutinoside (FC = 4.80), kaempferol-4’-O-glucoside (FC = 3.64), and ferulic acid (FC = 9.09).

In terms of flavor-related metabolites, the JX group exhibited a significant accumulation of key aroma-active compounds. A detailed list of these flavor-associated differential metabolites (with confidence levels Level 1 and 2) is presented in Table S4. These included terpenoids/norisoprenoids like macrophypene B (FC = 5.06), spicy components such as 6-gingerol (FC = 4.95), and floral aroma compounds like methyl jasmonate (FC = 4.03) and phenethyl alcohol (FC = 8.45). This collective shift in the metabolite profile provides a chemical basis for the complex, rich flavor characteristics developed through the roasting and sealed aging process.

To further confirm the reliability of metabolite identification, six flavor-related differential metabolites (trans-cinnamate, [6]-gingerol, vanillate, isoscopoletin, *L*-phenylalanine, and L-leucine) were verified by comparing their fragment ion patterns with authentic standard references using mirror plot analysis (Fig. S4). The matching fragment patterns between standards and experimental samples confirmed the successful identification of these six metabolites, providing robust evidence that these compounds are closely associated with flavor formation in the processed jujube products.

#### 3.4.4 KEGG pathway enrichment analysis

Functional category analysis based on the KEGG database was performed to annotate and classify the observed chemical changes (Fig. 5c; Table S3). It should be noted that these enrichments do not imply any enzymatic activity or biological regulation, as the high-temperature treatment completely inactivates cellular enzymes and causes cell death. Instead, they serve as a heuristic tool to annotate and categorize the differential metabolites, reflecting the chemical transformations resulting from thermal processing and sealed storage. The differential metabolites were significantly enriched in several key chemical categories, including phenylpropanoid biosynthesis-related compounds, flavonoid biosynthesis-related compounds, various amino acid-related chemical transformations (e.g., tryptophan, tyrosine, and phenylalanine chemistry), lipid chemical transformations (linoleic acid chemistry, alpha-linolenic acid chemistry), and plant hormone-related compounds.

The enrichment of phenylpropanoid and flavonoid biosynthesis-related compounds aligns with the observed upregulation of corresponding metabolites, indicating the chemical formation of these compounds during thermal processing. The changes in amino acid-related chemicals, coupled with the downregulation of free amino acids, reflects their chemical consumption in non-enzymatic reactions such as Maillard and Strecker degradation under the combined treatment. Additionally, changes in lipid-related chemicals suggest alterations in lipid chemical composition due to thermal degradation. In total, over 150 differential metabolites were annotated to these significantly altered chemical categories, underscoring the broad scope of non-enzymatic chemical transformations induced by the roasting and sealing process, which collectively contributes to the altered nutritional and functional properties of the final product.

## 4 Discussion

The physiological and biochemical results clearly showed the distinct outcomes among the four treatments. The control (CK) displayed rapid senescence (Fig. 1). Sealed storage alone (CK-S) gave moderate preservation, likely via a modified atmosphere that suppresses respiratory metabolism (Wang et al., 2025). In contrast, dry-heat roasting alone (CK-R) induced immediate changes, such as a sharp rise in 5-HMF and a transient increase in FRAP, TPC, and TFC on day 1 (Fig. 2, Fig. 3a) (Agila & Barringer, 2012). However, CK-R suffered the fastest depletion of vitamin C, which is primarily attributed to the thermal lability of ascorbic acid that rapidly degrades under high-temperature processing through oxidative and hydrolytic reactions (Lee & Kader, 2000), and Maillard precursors (Fig. 3b,c), resulting in the hardest product (32.7 N) among all groups. Neither single treatment achieved the desired balance between aroma development and quality preservation. The combined treatment (JX) is thus necessary to integrate the aroma-generating potential of roasting with the preservation benefit of sealed storage, achieving synergistic effects that neither treatment could accomplish alone.

**Fig. 6:**
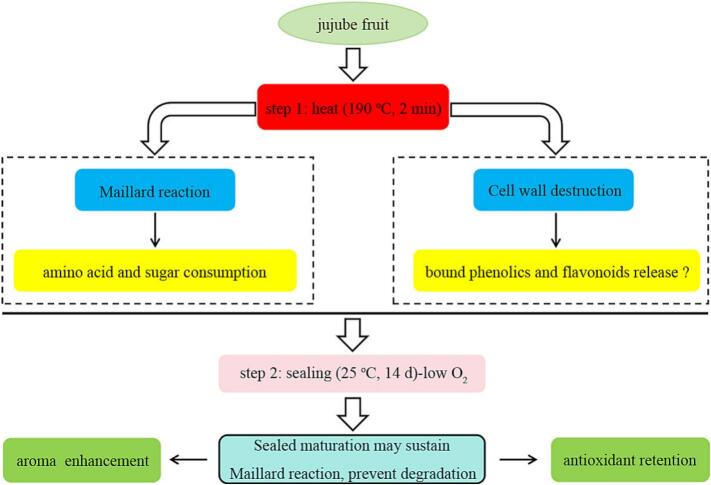
Schematic hypothesis diagram showing the “roasting-initiation + sealed-aging amplification” two-stage synergistic model. Stage I (190 °C, 2 min) triggers Maillard reaction and releases bound phenolics/flavonoids. Stage II (sealed storage, 14 days) creates a hypoxic environment that amplifies Maillard reaction while protecting antioxidants, ultimately resulting in intense roasted aroma and superior antioxidant capacity in the JX product. Details seen the text.

The combined treatment (JX) integrated and amplified the benefits of its components while reducing their drawbacks. JX gave the highest sensory score, better color and texture (Table 1), and the best preservation of FRAP, TPC, and TFC at day 14 (Fig. 2). Most importantly, JX showed the strongest Maillard signature: the highest 5-HMF accumulation and the greatest loss of free amino acids and reducing sugars (Fig. 3). This confirms that the initial roast triggered Maillard pathways and the sealed storage directed and amplified them. The conclusion is supported not only by 5-HMF data but also by precursor depletion and chemomic identification of many flavor-active compounds (see Supplementary Table S4). The anaerobic environment likely suppressed oxidative side reactions, allowing Maillard pathways to proceed more efficiently and accumulate advanced products like 5-HMF, a principle consistent with traditional Wu Zao processing and theoretical models of the Maillard reaction under limited oxygen availability (Van Boekel, 2006). Specifically, the Maillard reaction is known to produce 5-HMF under dry-heat conditions (Agila & Barringer, 2012; Taş & Gökmen, 2018). Beyond merely preserving initial reaction products, the sealed storage created a controlled, anaerobic environment that facilitated a “maturation” or “aging” process, which is analogous to the post-fermentation aging of certain teas (e.g., Pu-erh) or the bottle aging of wines under reduced oxygen conditions (Furtado et al., 2021; Liu et al., 2022). This phase allowed slow, non-enzymatic transformations—including further Maillard reactions, Strecker degradation, and interactions among various flavor precursors—to harmonize the flavor profile. It also prevented the volatilization and oxidative degradation of volatile aroma compounds generated during roasting, transforming sharp, initial roasted notes into the characteristic, well-rounded, and rich roasted aroma of the final product. Thus, sealing actively guided the chemical trajectory initiated by heat toward a superior sensory endpoint. A schematic diagram illustrating this “roasting-initiation + sealed-aging amplification” two-stage synergistic model is presented in Fig. 6.

The distinct aroma profile of JX stems fundamentally from the employed thermal processing method. Traditional Wu Zao primarily utilizes repeated cycles of drying and smoking dry-heat treatment, whereas our protocol applies a brief, high-temperature dry-heat roast. This dry-heat approach typically promotes more intense Maillard reactions and pyrolysis, leading to a broader and more intense spectrum of volatile compounds associated with roasted, nutty, and caramel-like aromas, which likely explains the perceived stronger fragrance compared to traditional Wu Zao (Table S2). Roasting significantly affects sensory qualities, color, physicochemical components, and identification of key aroma compounds in food products (Ye et al., 2025). The softer, more glutinous texture and higher sweetness of traditional Wu Zao can likely be attributed to its complex processing method, which includes repeated cycles of low-temperature (35–45 °C) drying and smoking as well as an initial blanching step (90–100 °C for 3–5 min). In contrast, our simplified single-step high-temperature roasting (190 °C, 2 min) results in a firmer product with a different textural profile. Nonetheless, the JX product demonstrates notable advantages over traditional Wu Zao, including significantly stronger aromatic intensity and superior accumulation of antioxidant nutrients (Table S2, Fig. 2). This represents a significant process innovation, offering a streamlined alternative that is more amenable to standardization and industrial scale-up.

The chemomic results support our hypothesis. Large chemical changes were found in JX (Figs. S2, S3), explaining the quality improvements. To ensure reliability, our discussion focuses primarily on compounds with confidence levels Level 1 and Level 2. First, the treatment strongly promoted the accumulation of flavonoids and phenolic acids (e.g., apigenin-7-O-rutinoside, ferulic Acid), corroborating the higher TPC and TFC and explaining the sustained antioxidant capacity (FRAP). Notably, examination of the top 30 differentially accumulated metabolites (Table 2) revealed that the majority are antioxidant-related compounds, including various flavonoids and phenolic acids, further corroborating the enhanced antioxidant capacity in JX. Specifically, isorhamnetin-3-O-glucoside was significantly up-regulated (FC = 4.42), directly contributing to the enhanced antioxidant capacity. KEGG enrichment analysis revealed an enrichment of compounds annotated to phenylpropanoid/flavonoid pathways (Fig. 5), consistent with non-enzymatic chemical changes in heat-treated foods (Liu et al., 2025). Second, free amino acids (e.g., l-glutamine, *L*-phenylalanine) were dramatically down-regulated, confirming their consumption as Maillard and Strecker reaction precursors through non-enzymatic chemical conversions (Liu et al., 2022; Yu et al., 2021).

Chemomics identified specific flavor-active compounds enriched in JX (Supplementary Table S4). The increase in compounds such as 6-gingerol (spicy), macrophypene B (floral/fruity), methyl jasmonate (floral), and phenethyl alcohol (rosy) provides a direct chemical basis for the complex sensory profile. These findings are consistent with previous studies demonstrating that roasting temperature significantly influences flavor characteristics in jujube-based products (Li et al., 2025). The up-regulation of phenolic acids like ferulic acid (FC = 9.09), which can yield smoky notes upon decarboxylation (Belviso et al., 2013), contributes to flavor complexity. Phenolic additives can affect physicochemical properties and antioxidant activity of blackened jujube (Song et al., 2023). The enrichment of specific terpenoids/norisoprenoids suggests dry-heat roasting promoted carotenoid degradation into potent aroma-active compounds (He et al., 2025). Furthermore, alterations in lipid and hormone-related compounds (Fig. 5) suggest broader chemical transformations.

The chemomic evidence reveals the distinctive chemical signature of the “dry-heat–sealed aging” process: non-enzymatic formation of protective phenolics, chemical conversion of primary metabolites into flavor-generating products, and directional accumulation of diverse positive aroma compounds. This integrated chemical transformation establishes the unique biochemical trajectory culminating in a superior product.

This study provides key innovations in fruit postharvest processing. While previous research focused on single treatments—either heat (Morgado et al., 2016; Rodov et al., 2000) or sealed storage (Soltani et al., 2015; Zhang et al., 2015)—our work is the first to deconstruct “Wu Zao” processing (Wu et al., 2025) into a two-step protocol: dry-heat roasting followed by sealed anaerobic aging. This validates our hypothesis: the combined treatment actively transforms the fruit through non-enzymatic chemical reactions, not just preserves it. The sealed environment directs chemical pathways initiated by dry-heat, creating a novel “aroma-enhanced jujube.” From a practical view, this simplified method is less labor-intensive than traditional Wu Zao, offering potential for efficient industrial production.

Several limitations should be noted: (1) The chemomic analysis compared only JX vs. CK to focus on the holistic impact of the integrated treatment. The physiological and biochemical data demonstrated the superiority of the combined treatment. (2) High-temperature processing reduced vitamin C, but this was offset by higher flavonoids, phenolics, and roasting compounds (e.g., 5-HMF). The nutritional profile is altered rather than simply degraded. (3) Only one cultivar and fixed parameters were used. Future work should optimize conditions for other varieties and employ chemical analytical approaches to elucidate transformation networks. Detailed flavoromics (GC–MS/O) is needed to link identified compounds to sensory attributes (He et al., 2025; Zhan et al., 2023). A direct sensory comparison between JX and traditional Wu Zao would also be valuable.

## 5 Conclusions

This study developed a simplified two-step processing protocol (roasting-sealing) inspired by traditional “Wu Zao” production. The combined treatment (JX) achieved the highest sensory acceptability and best-preserved cellular integrity, with significantly enhanced bioactive compound accumulation including total phenolics and flavonoids compared to the untreated control after 14 days. Intense Maillard reaction was confirmed in JX, as evidenced by maximal 5-HMF accumulation, together with the greatest depletion of free amino acids and reducing sugars. Metabolomic analysis identified numerous differential metabolites, revealing up-regulation of flavor-active compounds that provide a chemical basis for the enhanced roasted aroma. Limitations: (1) JX shows reddish instead of black color, with slightly lower overall acceptability score yet stronger aroma and higher antioxidant capacity compared to traditional Wu Zao; (2) the transformation mechanism remains to be clarified, but the simplified process greatly reduces processing time; (3) more comparative studies with traditional Wu Zao are needed. This work establishes a simplified, controllable, and industrially viable processing paradigm that transforms fresh jujube into a flavor-rich, nutritionally enhanced product, offering a modern pathway for value-added fruit processing.

## CRediT authorship contribution statement

**Shan Tian:** Writing – original draft, Software, Resources, Methodology, Investigation, Formal analysis. **Yudie Yao:** Visualization, Validation, Investigation, Data curation. **Changlai Liu:** Visualization, Validation, Investigation, Data curation. **Benliang Deng:** Writing – review & editing, Supervision, Project administration, Conceptualization.

## Ethical statement

The sensory evaluation was conducted with a trained panel (*n* = 10) of healthy adult volunteers from the Department of Food Science. Because this study involved only the tasting of a conventional food product (rozed jujube) and did not include any clinical interventions or collection of sensitive personal information, formal ethical approval was not required. All participants provided written informed consent prior to the evaluation. No personal identifiers of the participants are disclosed in this manuscript.

## Declaration of generative AI and AI-assisted technologies in the writing process

During the preparation of this work the author used AI service in order to improve the language quality. After using this tool/service, the author(s) reviewed and edited the content as needed and take(s) full responsibility for the content of the publication.

## Funding

This work was supported by grants from the Opening Topic Foundation of “China-Loess Plateau Water Loss and Soil Erosion” Process and Control Key Laboratory in Ministry of Water Conservancy [grant number 190412] and Henan Provincial Natural Science Foundation (grant number 262300420472).

## Declaration of competing interest

The authors declare that they have no known competing financial interests or personal relationships that could have appeared to influence the work reported in this paper.

## Data Availability

The authors confirm that the data supporting the findings of this study are available within the article. Further inquiries can be directed to the corresponding author.

## Appendix A Supplementary data

Supplementary material Figure S1. Metabolomic comparison between CK and JX groups. Figure S2. Multivariate analysis of metabolomic profiles between CK and JX groups. Figure S3. VIP-based volcano plot of differentially abundant metabolites between JX and CK groups. Figure S4. Mirror plot verification of six flavor-related differential metabolites identified in this study.
