# Supplementary material for: From complexity to simplicity: a traditional-inspired roasting-sealing process enhances jujube aroma and antioxidant properties
Source: Food Chem X. 2026 Jun 15;37:104109. doi: 10.1016/j.fochx.2026.104109 (PMC13293758; doi:10.1016/j.fochx.2026.104109)
Supplement: Supplementary material — Figure S1. Metabolomic comparison between CK and JX groups. Figure S2. Multivariate analysis of metabolomic profiles between CK and JX groups. Figure S3. VIP-based volcano plot of differentially abundant metabolites between JX and CK groups. Figure S4. Mirror plot verification of six flavor-related differential metabolites identified in this study. [file mmc1.zip › mmc1/Suppl Mater revised/Table S1 sample number.docx]

**Table S1** Sample identification and treatment groups for metabolomic analysis.

| Sample tissue | Process description | Sample name | Groups |
| --- | --- | --- | --- |
| Jujube fruit | No processing | CK-1 | CK (control) |
| Jujube fruit | No processing | CK-2 | CK (control) |
| Jujube fruit | No processing | CK-3 | CK (control) |
| Jujube fruit | Roasting+sealing | JX-1 | JX (roasting-sealing Jujube) |
| Jujube fruit | Roasting+sealing | JX-2 | JX (roasting-sealing Jujube) |
| Jujube fruit | Roasting+sealing | JX-3 | JX (roasting-sealing Jujube) |
