# Supplementary material for: From complexity to simplicity: a traditional-inspired roasting-sealing process enhances jujube aroma and antioxidant properties
Source: Food Chem X. 2026 Jun 15;37:104109. doi: 10.1016/j.fochx.2026.104109 (PMC13293758; doi:10.1016/j.fochx.2026.104109)
Supplement: Supplementary material — Figure S1. Metabolomic comparison between CK and JX groups. Figure S2. Multivariate analysis of metabolomic profiles between CK and JX groups. Figure S3. VIP-based volcano plot of differentially abundant metabolites between JX and CK groups. Figure S4. Mirror plot verification of six flavor-related differential metabolites identified in this study. [file mmc1.zip › mmc1/Suppl Mater revised/Table S2 JX jujube vs smoked jujube.docx]

**Table S2 Comparison of selected characteristics between commercially available Wu Zao Jujube and JX products**Data are presented as mean ± SD (n = 3). Different letters indicate significant differences (p < 0.05, Duncan's test).

|  | JX product | Wu Zao (Yanan) | Wu Zao (Dingxi) | Wu Zao (Zhongwei) |
| --- | --- | --- | --- | --- |
| FRAP value | 8.7 ± 0.5a | 2.4 ± 0.3b | 2.8 ± 0.4b | 2.5 ± 0.2b |
| Aroma | very high | high | low | high |
| Color | red | black | black | black |
| Sweetness | low | high | moderate | high |
| Texture Hardness (N) | 20.9 ± 1.3a | 14.3 ± 0.9b | 16.1 ± 1.1b | 14.8 ± 0.8b |
| Overall Acceptability Score | 7.6 ± 0.2b | 8.4 ± 0.2a | 8.2 ± 0.3a | 8.3± 0.2a |
| Process complex | simple (2-step) | complex | complex | complex |
