# Supplementary material for: From complexity to simplicity: a traditional-inspired roasting-sealing process enhances jujube aroma and antioxidant properties
Source: Food Chem X. 2026 Jun 15;37:104109. doi: 10.1016/j.fochx.2026.104109 (PMC13293758; doi:10.1016/j.fochx.2026.104109)
Supplement: Supplementary material — Figure S1. Metabolomic comparison between CK and JX groups. Figure S2. Multivariate analysis of metabolomic profiles between CK and JX groups. Figure S3. VIP-based volcano plot of differentially abundant metabolites between JX and CK groups. Figure S4. Mirror plot verification of six flavor-related differential metabolites identified in this study. [file mmc1.zip › mmc1/Suppl Mater revised/Table S4 flavor metabolite.docx]

**Table S4 Potential flavor-related differential metabolites between JX and CK**

Widely targeted metabolomics was performed to identify flavor-active metabolites differentially accumulated between JX (roasting + sealed storage) and CK (control) after 14 days of storage. Metabolites were selected with VIP ≥ 1, FC ≥ 2 or ≤ 0.5, and P-value < 0.05 or FDR < 0.05. Only metabolites with MSI confidence levels of Level 1 (identified by authentic standards) or Level 2 (tentatively identified by MS/MS matching) were included. The table contains two sections: Aroma-Related Compounds (Rows 1–30) and Amino Acid-Derived Flavor Compounds (Rows 31–41).

| No. | Differentially Expressed Metabolite | FC | FDR | Level | Flavor trait |
| --- | --- | --- | --- | --- | --- |
| 1 | trans-Cinnamate | 18.68 | 0.0279 | 1 | Cinnamon, spicy |
| 2 | 3-beta,12-dihydroxy-13-methyl-podocarpane-8,10,13-tiene | 12.79 | 0.00535 | 1 | Woody, herbal |
| 3 | Isoscopoletin | 8.29 | 0.00299 | 1 | Coumarin-like, sweet |
| 4 | 5-hydroxy-1-(4-hydroxy-3-methoxyphenyl)decan-2-one | 6.09 | 0.00350 | 1 | Spicy, aromatic |
| 5 | 3-Hydroxybenzoate | 6.09 | 0.00094 | 1 | Mild aromatic |
| 6 | 6-Amino-1-naphthoic acid | 5.65 | 0.0107 | 1 | Mild aromatic |
| 7 | 13-(methoxymethyl)-10,11,11-trimethyltetracyclo[8.2.1.01,5.08,12]tridec-6-en-13-ol | 5.01 | 0.000059 | 1 | Woody, amber |
| 8 | [6]-Gingerol | 4.95 | 0.00227 | 1 | Gingerol, pungent spicy |
| 9 | 3-Methylbenzaldehyde | 4.76 | 0.00582 | 1 | Almond-like, aromatic |
| 10 | 10-hydroxy-4,6,8,10-tetramethyldodec-4-en-3-one | 4.75 | 0.0168 | 1 | Fruity, ketonic odor |
| 11 | 2-Hydroxy-4-methylpentanoic acid | 4.70 | 0.00234 | 1 | Milk-like, fruity |
| 12 | Benzoate | 4.71 | 0.0193 | 2 | Sweet, floral fragrance |
| 13 | Succinyladenosine | 4.62 | 0.00284 | 1 | Mild aromatic |
| 14 | Hispanolone | 4.68 | 0.00089 | 2 | Herbal, spicy aroma |
| 15 | 3-Acetylbenzoic acid | 4.06 | 0.00235 | 1 | Sweet, aromatic |
| 16 | (1a*S*,3a*R*,7a*S*,7b*S*)-1,1,7-trimethyl-1a,2,3,3a,4,5,7a,7b-octahydrocyclopropa[*a*]naphthalene | 3.77 | 0.0148 | 2 | Woody, amber notes |
| 17 | Vanillate | 3.42 | 0.00105 | 1 | Vanilla aroma |
| 18 | Ambroxide | 2.80 | 0.0358 | 1 | Ambergris, woody |
| 19 | 2,6-Dimethyl-2,5-heptadienoic acid | 2.62 | 0.0159 | 1 | Fruity, sour |
| 20 | 4-Hydroxy-3-methoxy-benzaldehyde | 2.55 | 0.00081 | 2 | Vanilla, sweet |
| 21 | 1-(4-hydroxy-3-methoxyphenyl)decan-5-yl acetate | 2.42 | 0.00620 | 1 | Fruity, floral |
| 22 | 5-Ethenyl-2-(2-Hydroxypropan-2-Yl)-4-(Prop-1-En-2-Yl)Cyclohexan-1-Ol | 2.26 | 0.00674 | 1 | Floral, herbal |
| 23 | 4-Hydroxybenzoate | 0.38 | 0.00115 | 2 | Mild aromatic |
| 24 | 4-Ethynylbenzaldehyde | 0.255 | 0.0174 | 1 | Mild aromatic |
| 25 | 7-Hydroxy-1,1,7-Trimethyl-Decahydro-1*H*-Cyclopropa[*E*]Azulen-2-one | 0.225 | 0.00043 | 1 | Camphor, herbal |
| 26 | 4-Hydroxybenzaldehyde | 0.24 | 0.00367 | 1 | Sweet, aromatic |
| 27 | (+)-exo-5-Hydroxycamphor | 0.069 | 0.0291 | 1 | Camphor-like |
| 28 | 1-(hydroxymethyl)-7,7-dimethylbicyclo[2.2.1]heptan-2-one | 0.086 | 0.00384 | 1 | Camphoraceous |
| 29 | 2-Hydroxy-2-(Hydroxymethyl)-6,6-Dimethylbicyclo[3.1.1]Heptan-3-one | 0.056 | 0.0291 | 1 | Camphor-like, herbal |
| 30 | 2-Hydroxyphenylacetate | 0.017 | 0.00340 | 1 | Phenolic, sweet |
| 31 | L-Leucine | 0.288 | 0.00578614 | 1 | Bitterness, milky aroma |
| 32 | L-Norleucine | 0.396 | 0.00635933 | 1 | Slight bitterness |
| 33 | L-Isoleucine | 0.398 | 0.009363552 | 1 | Slight bitterness, cheesy note |
| 34 | D-allo-Isoleucine | 0.395 | 0.019658539 | 1 | Slight bitterness |
| 35 | L-Asparagine | 2.079 | 0.023705918 | 1 | Mild sweet and umami taste; contributes to freshness |
| 36 | L-Tyrosine | 0.434 | 0.008878336 | 1 | Bitterness, slight sweetness, cheesy flavor |
| 37 | L-Phenylalanine | 0.351 | 0.001919381 | 1 | Strong bitterness, sweet floral aroma |
| 38 | L-Valine | 0.153 | 0.001730018 | 1 | Slight bitterness, chocolate-like note |
| 39 | L-Lysine | 0.185 | 0.000515882 | 1 | Slight bitterness, slightly sweet |
| 40 | L-Arginine | 0.234 | 0.002336281 | 1 | Slight bitterness, slight sweet aftertaste |
| 41 | Tryptophan | 0.322 | 0.007410653 | 1 | Mild bitterness, floral aroma |
