# Supplementary figures and images for: From complexity to simplicity: a traditional-inspired roasting-sealing process enhances jujube aroma and antioxidant properties
Source: Food Chem X. 2026 Jun 15;37:104109. doi: 10.1016/j.fochx.2026.104109 (PMC13293758; doi:10.1016/j.fochx.2026.104109)

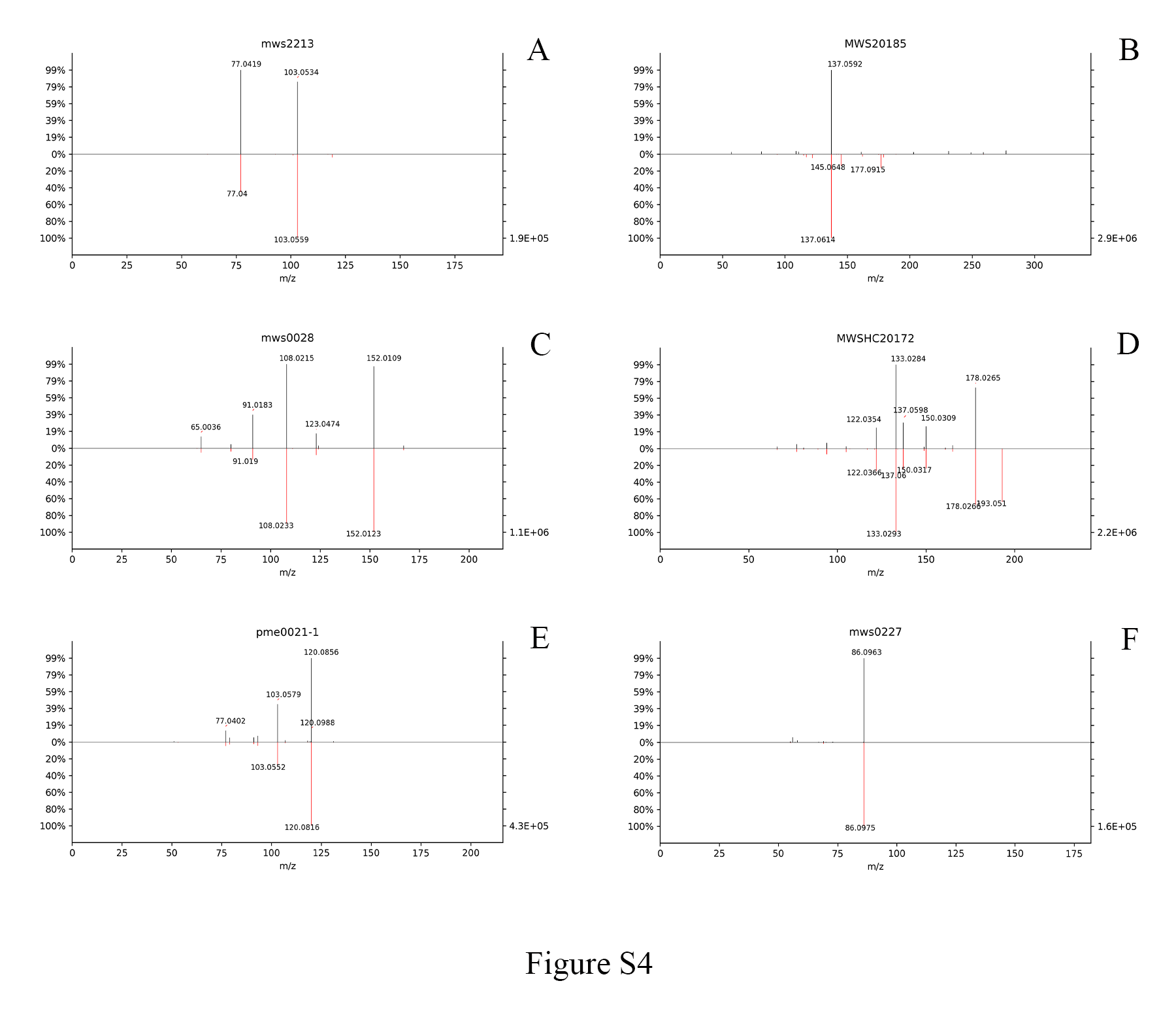

Supplement: Supplementary material — Figure S1. Metabolomic comparison between CK and JX groups. Figure S2. Multivariate analysis of metabolomic profiles between CK and JX groups. Figure S3. VIP-based volcano plot of differentially abundant metabolites between JX and CK groups. Figure S4. Mirror plot verification of six flavor-related differential metabolites identified in this study. [file mmc1.zip › mmc1/Suppl Mater revised/Figure S4 new.tif]

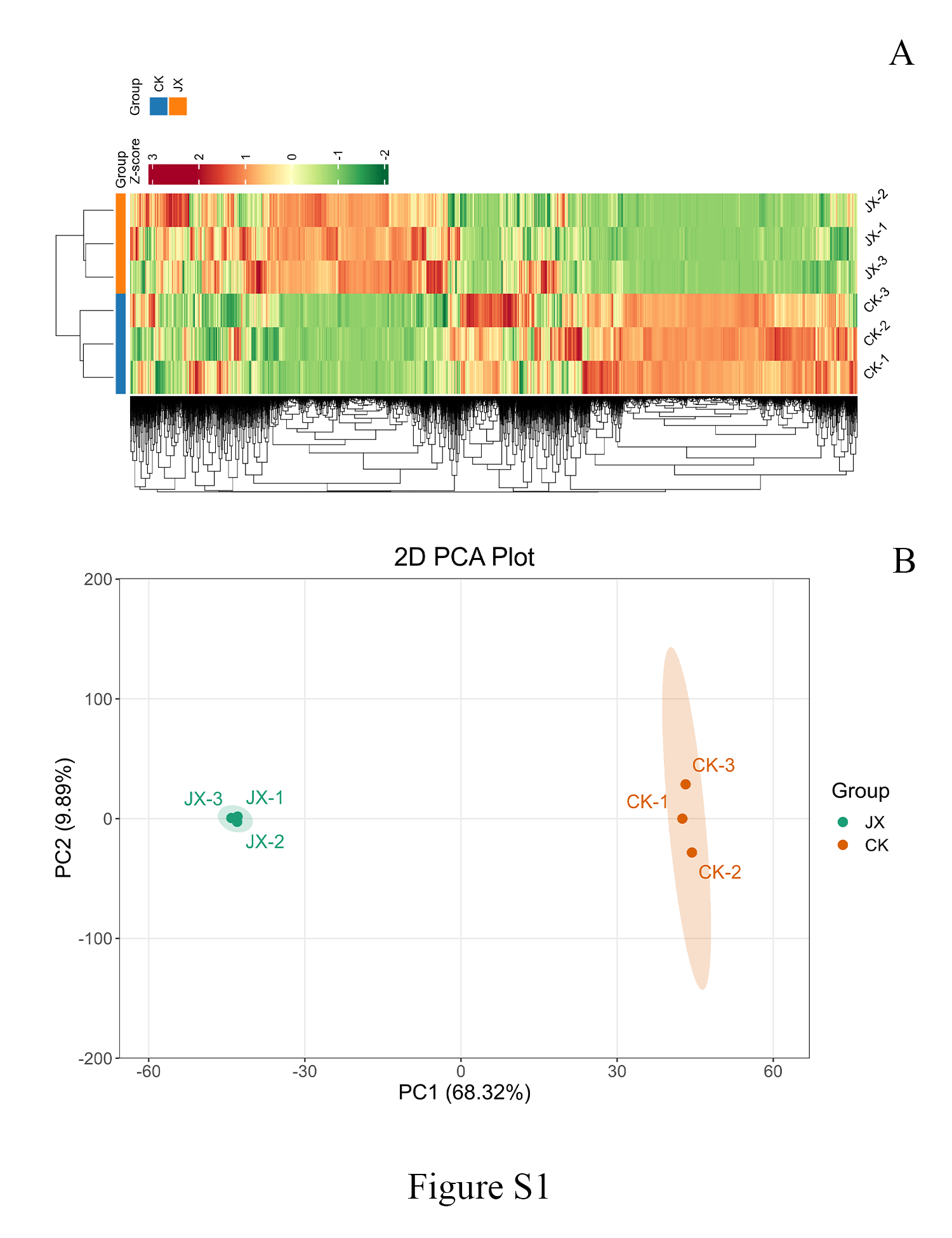

Supplement: Supplementary material — Figure S1. Metabolomic comparison between CK and JX groups. Figure S2. Multivariate analysis of metabolomic profiles between CK and JX groups. Figure S3. VIP-based volcano plot of differentially abundant metabolites between JX and CK groups. Figure S4. Mirror plot verification of six flavor-related differential metabolites identified in this study. [file mmc1.zip › mmc1/Suppl Mater revised/Figure S1 revised.tif]

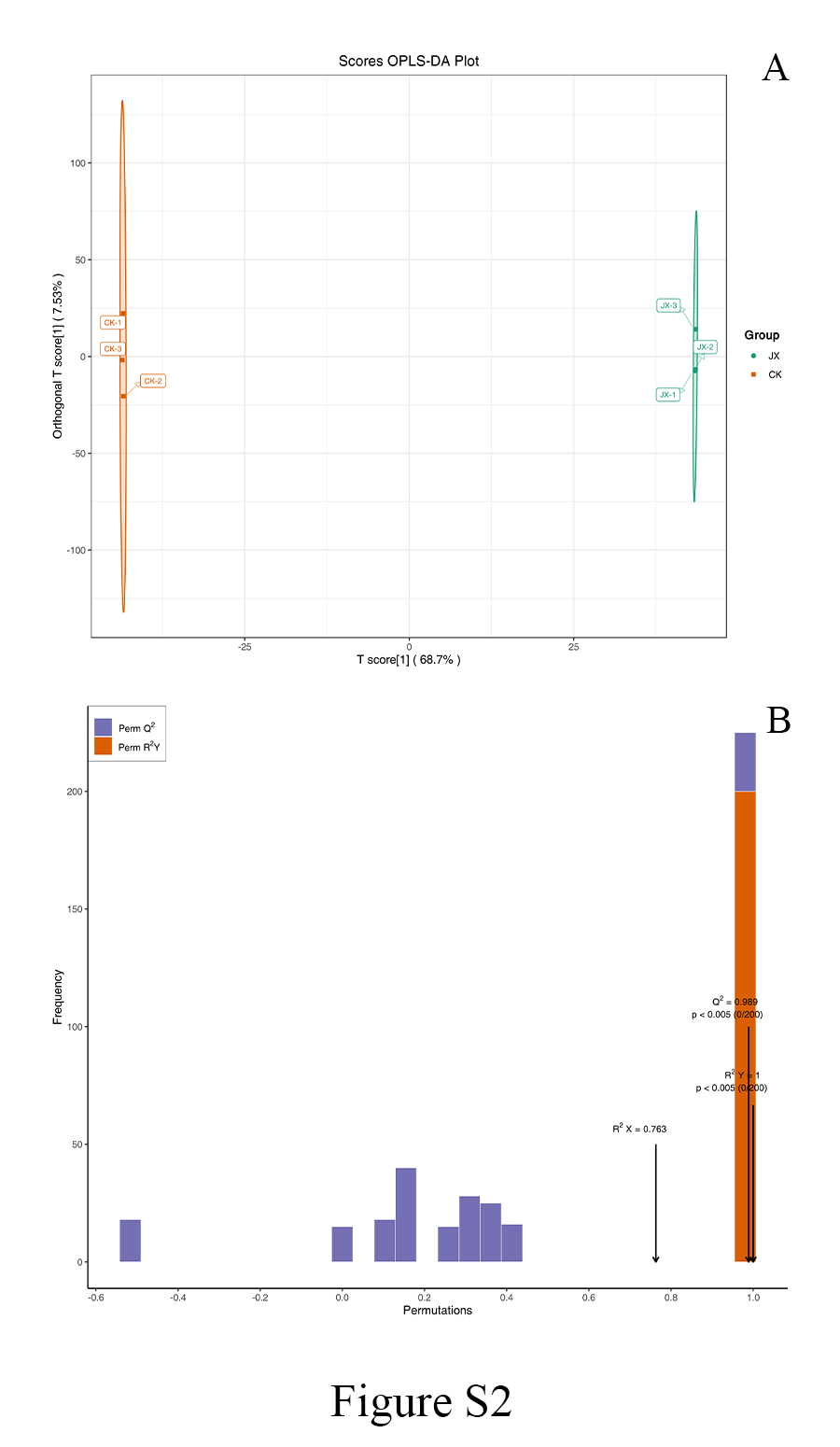

Supplement: Supplementary material — Figure S1. Metabolomic comparison between CK and JX groups. Figure S2. Multivariate analysis of metabolomic profiles between CK and JX groups. Figure S3. VIP-based volcano plot of differentially abundant metabolites between JX and CK groups. Figure S4. Mirror plot verification of six flavor-related differential metabolites identified in this study. [file mmc1.zip › mmc1/Suppl Mater revised/Figure S2 revised.tif]

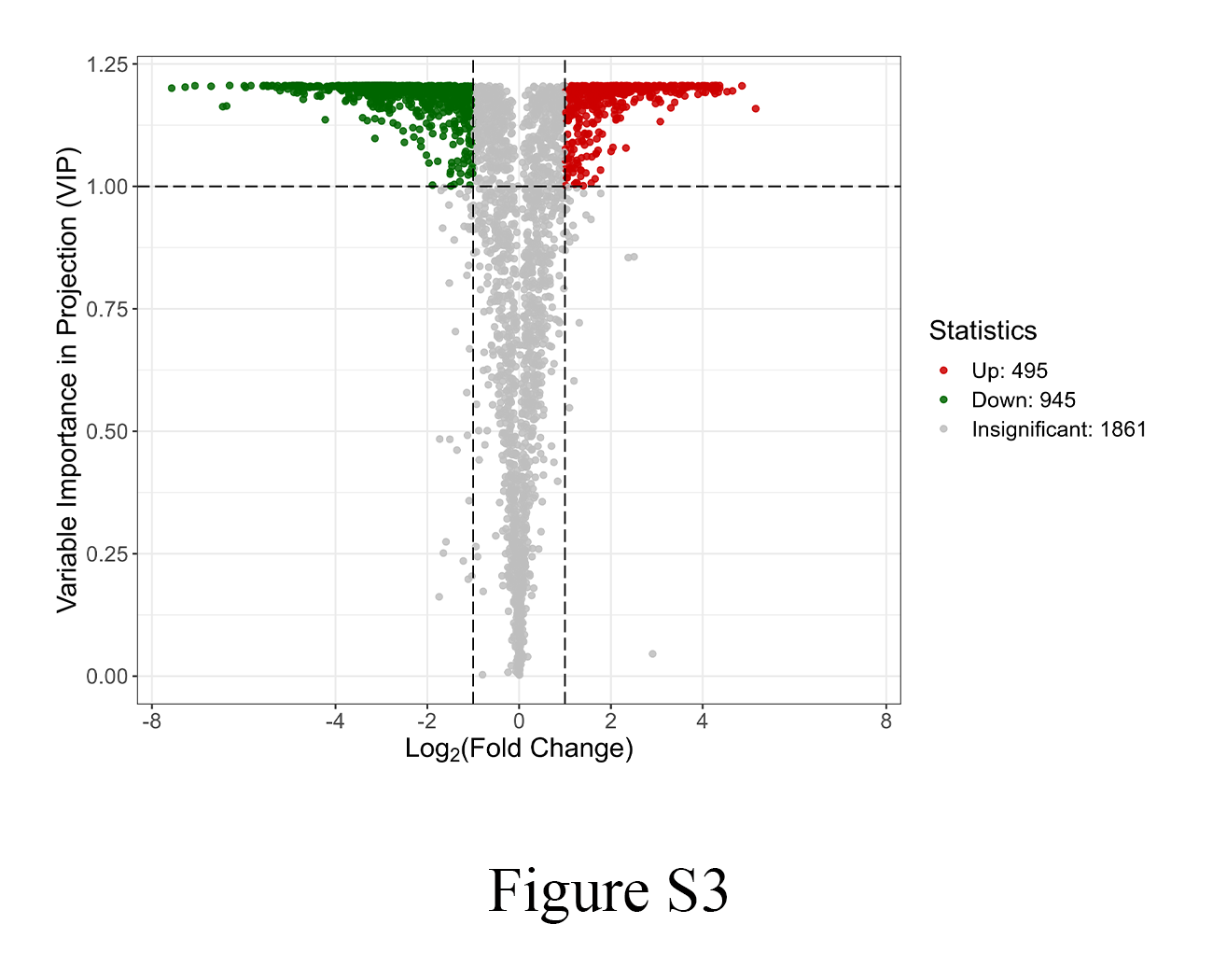

Supplement: Supplementary material — Figure S1. Metabolomic comparison between CK and JX groups. Figure S2. Multivariate analysis of metabolomic profiles between CK and JX groups. Figure S3. VIP-based volcano plot of differentially abundant metabolites between JX and CK groups. Figure S4. Mirror plot verification of six flavor-related differential metabolites identified in this study. [file mmc1.zip › mmc1/Suppl Mater revised/Figure S3 Revised.tif]
